# Editorial: The role of communication and emotion in human-robot interaction: a psychological perspective

**DOI:** 10.3389/frobt.2026.1897548

**Published:** 2026-06-29

**Authors:** Karolina Eszter Kovács, Péter Korondi, Mihoko Niitsuma, Yousef Ibrahim, Csilla Csukonyi, Balázs Őrsi

**Affiliations:** 1 Institute of Psychology, Faculty of Humanities, University of Debrecen, Debrecen, Hungary; 2 Department of Mechatronics, Faculty of Engineering, University of Debrecen, Debrecen, Hungary; 3 Department of Precision Mechanics, Chuo University, Tokyo, Japan; 4 Centre for Smart Analytics, Federation University Australia, Ballarat, VIC, Australia

**Keywords:** communication, human-robot collaboration, human-robot interaction (HRI), industry 5.0, robotic psychology, trust

Research in human–robot interaction increasingly demonstrates that communication and emotion are not peripheral aspects of social robotics, but fundamental conditions for it. The six contributions in this Research Topic consistently approach this Research Topic from a psychological perspective and collectively show that human interpretations, impressions, and behaviours toward robots strongly depend on how information is communicated, through which channels, in what contexts, and with what social meaning. An important strength of the Research Topic is that it examines these questions beyond a single narrow laboratory setting, extending to transportation infrastructure and expo environments, mental health screening, early childhood development, and the broader field of multimodal affective robotics.

Adachi and Kakio investigated how communication embedded into building infrastructure can support smoother interactions between humans and service robots in elevator environments. The authors developed a Human-Facility Interaction (HFI) system that provided passengers with verbal and nonverbal information while they shared an elevator with a service robot. In two hotel-based experiments involving 31 participants, voice announcements significantly improved impressions of both the robot and the elevator while also reducing perceived waiting time. In contrast, light-based communication cues showed more context-dependent effects. The study highlights the importance of socially aware infrastructure in facilitating human acceptance of robots in public environments.

Fujii et al. explored how different instruction styles influence people’s perceptions of android robot facial expressions. Using the android robot Nikola, capable of producing 63 nuanced facial expressions, the researchers conducted a large-scale field experiment at Expo 2025 in Japan with more than 600 participants. The findings showed that abstract user instructions led participants to evaluate the robot’s emotional responses as more appropriate and expressive than explicit emotion-related instructions. The study suggests that users actively contribute to emotion attribution in HRI and that interpretive ambiguity may enhance perceived social agency in robots.

Tsvetkova examined perceptions of the Furhat social robot in a psychological assessment context using a pilot mixed-method design. Young adult participants interacted with the robot while it administered the DASS-21 mental health screening questionnaire and subsequently evaluated the robot’s warmth, competence, and discomfort levels using the RoSAS-SF scale. Participants generally perceived the robot as competent, relatively warm, and minimally discomforting, while qualitative feedback emphasised openness, trust, and the value of a non-judgmental interactional atmosphere. At the same time, the study identified limitations in speech recognition and restricted nonverbal expressivity, pointing to important future directions for mental health-oriented HRI systems.

Chengxing et al. investigated how colour lightness influences emotional arousal in the digital facial expressions of social robots. Through experimental studies in which robot faces displayed emotions at different lightness levels, the authors demonstrated that lightness alone significantly modulates perceived emotional intensity. Higher lightness levels tended to support more positive impressions, whereas lower lightness enhanced perceptions of negative emotional arousal. The study contributes to affective HRI research by showing that subtle visual micro-design features, beyond categorical facial expressions themselves, can shape emotional communication between humans and robots.

Ishibashi et al. explored whether praise delivered by a social robot could enhance task persistence in toddlers aged 18–24 months. Using the child-like social robot CommU, the authors compared praise delivered by a robot and by a human agent during a persistence task. Children in the praise condition demonstrated significantly greater persistence regardless of whether the praise came from the robot or the human. Additionally, the amount of time children spent visually attending to the agent was positively associated with persistence. The findings suggest that robot-delivered social feedback may already influence motivation and engagement during very early developmental stages.

Howcroft et al. conducted a scoping review on speech–touch integration in affective human–robot interaction. Reviewing 11 HRI implementations, the authors examined how spoken language and tactile interaction are coordinated in social robots and what effects these multimodal interactions have on users. Across comparative studies, the combination of speech and touch often enhanced perceptions of empathy, care, attachment, and calming compared to speech-only or touch-only interaction. The review also highlighted the importance of warm, soft, and naturalistic tactile experiences, while emphasizing the substantial psychological, cultural, and engineering challenges involved in designing robots capable of meaningful affective touch interaction.

The articles in this Research Topic collectively highlight three major insights regarding communication and emotion in human–robot interaction. First, social HRI communication emerges as fundamentally multimodal. Adachi and Kakio demonstrated that voice-based information embedded in building infrastructure can improve impressions of robots and reduce the subjective burden of waiting, whereas the effects of light-based communication are more context-dependent. Similarly, Chengxing et al. showed that even subtle visual micro-design features, such as colour lightness in digital facial expressions, systematically shape perceived emotional arousal. Expanding on this perspective, Howcroft et al. found that integrating speech and touch may enhance empathy, attachment, and a sense of calm, particularly when tactile interaction feels warm, soft, and naturalistic.

A second shared finding is that emotion in HRI is not simply transmitted by robots but co-constructed through human interpretation. Fujii et al. showed that abstract instructions increased perceived emotional appropriateness and expressiveness of robot behaviour, suggesting that users actively participate in constructing robot agency. Likewise, Tsvetkova found that a calm and non-judgmental robotic presence may support openness in mental health screening contexts.

Finally, the Research Topic demonstrates that communication and emotion influence not only impressions but also behaviour. Ishibashi et al. showed that praise delivered by a robot increased task persistence even in toddlers, while other studies addressed outcomes such as hesitation, emotional regulation, attachment, and self-disclosure. Together, the findings indicate that communication and emotion in HRI simultaneously shape perceptual, affective, and behavioural processes.

The Research Topic also points toward several directions for future research. Some studies relied on relatively small or culturally homogeneous samples, and many interactions were limited to short-term settings. In addition, challenges related to speech recognition, timing, materiality, and interactional robustness remained visible in several multimodal systems (Adachi and Kakio; Howcroft et al.; Tsvetkova). These limitations suggest the importance of future studies with greater ecological validity, broader cultural and developmental perspectives, and more stable interaction designs. At the same time, the present Research Topic already contributes meaningfully to the psychological understanding of social robotics by emphasising that communication, emotional meaning, and human interpretation continuously shape one another during human–robot interaction.

